# (1*R*,2*R*)-*N*,*N*′-Bis[1-(2-pyrid­yl)ethyl­idene]cyclo­hexane-1,2-diamine

**DOI:** 10.1107/S1600536810013607

**Published:** 2010-04-17

**Authors:** Muhammad Saleh Salga, Hamid Khaledi, Hapipah Mohd Ali, Rustam Puteh

**Affiliations:** a Department of Chemistry, University of Malaya, 50603 Kuala Lumpur, Malaysia; b Department of Physics, University of Malaya, 50603 Kuala Lumpur, Malaysia

## Abstract

In the title compound, C_20_H_24_N_4_, the cyclo­hexane ring adopts a chair conformation with the two imine groups linked at equatorial positions. The two halves of the mol­ecule are related by a crystallographic twofold rotation axis. The dihedral angle between the pyridine rings is 75.73 (3)°.

## Related literature

For the crystal structures of some Schiff bases derived from cyclo­hexane-1,2-diamine, see: Aslantaş *et al.* (2007 ▶); Glidewell *et al.* (2005 ▶); Liu *et al.* (2006 ▶).
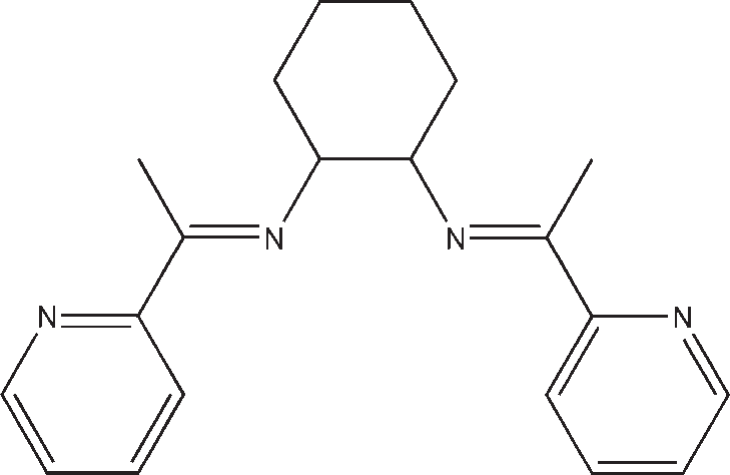


## Experimental

### 

#### Crystal data

C_20_H_24_N_4_
*M* *_r_* = 320.43
Monoclinic, 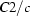

*a* = 18.0605 (3) Å
*b* = 8.9371 (1) Å
*c* = 11.1076 (2) Å
β = 97.970 (1)°
*V* = 1775.54 (5) Å^3^
*Z* = 4
Mo *K*α radiation
μ = 0.07 mm^−1^
*T* = 100 K
0.49 × 0.37 × 0.35 mm

#### Data collection

Bruker APEXII CCD diffractometer
Absorption correction: multi-scan (*SADABS*; Sheldrick, 1996 ▶) *T*_min_ = 0.965, *T*_max_ = 0.975
8186 measured reflections
2044 independent reflections
1833 reflections with *I* > 2σ(*I*)
*R*_int_ = 0.019

#### Refinement

*R*[*F* ^2^ > 2σ(*F* ^2^)] = 0.040
*wR*(*F* ^2^) = 0.121
*S* = 1.06
2044 reflections
110 parameters
H-atom parameters constrained
Δρ_max_ = 0.33 e Å^−3^
Δρ_min_ = −0.23 e Å^−3^

### 

Data collection: *APEX2* (Bruker, 2007 ▶); cell refinement: *SAINT* (Bruker, 2007 ▶); data reduction: *SAINT*; program(s) used to solve structure: *SHELXS97* (Sheldrick, 2008 ▶); program(s) used to refine structure: *SHELXL97* (Sheldrick, 2008 ▶); molecular graphics: *Mercury* (Macrae *et al.*, 2008 ▶); software used to prepare material for publication: *publCIF* (Westrip, 2010 ▶).

## Supplementary Material

Crystal structure: contains datablocks I, global. DOI: 10.1107/S1600536810013607/pv2274sup1.cif

Structure factors: contains datablocks I. DOI: 10.1107/S1600536810013607/pv2274Isup2.hkl

Additional supplementary materials:  crystallographic information; 3D view; checkCIF report

## Figures and Tables

**Fig. 1.**
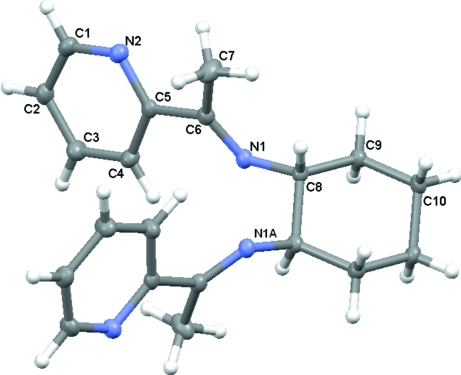
Thermal ellipsoid plot of the title compound at 50% probability level; hydrogen atoms are drawn as spheres of arbitrary radius. Symmetry code for the unlabeled atoms: -x, y, -z+3/2.

## supplementary crystallographic information

### Comment

The stucture of the title compound is presented in Fig. 1. The cyclohexane ring adopts a chair conformation with the two imines linked at equatorial positions. The two halves of the molecule are realted by a two-fold rotation. The dihedral angel between the two pyridine rings is 75.73 (3)°. The crystal structure is devoid of any inter- or intra- molecular interactions.

The bond distances and angles in the title molecule are in agreement with the corresponding bond distances and angles reported in some related structures (Aslantaş *et al.*, 2007; Glidewell *et al.*, 2005; Liu *et al.*, 2006).

### Experimental

A mixture of 2-acetylpyiridine (0.444 g, 4 mmol) and 1,2-diaminocyclohexane (0.224, 2 mmol) was refluxed in ethanol (50 ml) for 2 hours. The solution was then set aside overnight whereupon the yellow crystals of the title compound were formed.

### Refinement

Hydrogen atoms were placed at calculated positions (C—H 0.95-1.00 Å), and were treated as riding on their parent atoms with U_iso_(H) set to 1.2-1.5 U_eq_(C).

### Crystal data

**Table:**

C_20_H_24_N_4_	*F*(000) = 688
*M**_r_* = 320.43	*D*_x_ = 1.199 Mg m^−^^3^
Monoclinic, *C*2/*c*	Mo *K*α radiation, λ = 0.71073 Å
Hall symbol: -C 2yc	Cell parameters from 4367 reflections
*a* = 18.0605 (3) Å	θ = 2.3–30.3°
*b* = 8.9371 (1) Å	µ = 0.07 mm^−^^1^
*c* = 11.1076 (2) Å	*T* = 100 K
β = 97.970 (1)°	Block, pale yellow
*V* = 1775.54 (5) Å^3^	0.49 × 0.37 × 0.35 mm
*Z* = 4	

### Data collection

**Table:**

Bruker APEXII CCD diffractometer	2044 independent reflections
Radiation source: fine-focus sealed tube	1833 reflections with *I* > 2σ(*I*)
graphite	*R*_int_ = 0.019
φ and ω scans	θ_max_ = 27.5°, θ_min_ = 2.3°
Absorption correction: multi-scan (*SADABS*; Sheldrick, 1996)	*h* = −23→22
*T*_min_ = 0.965, *T*_max_ = 0.975	*k* = −11→11
8186 measured reflections	*l* = −14→14

### Refinement

**Table:**

Refinement on *F*^2^	Primary atom site location: structure-invariant direct methods
Least-squares matrix: full	Secondary atom site location: difference Fourier map
*R*[*F*^2^ > 2σ(*F*^2^)] = 0.040	Hydrogen site location: inferred from neighbouring sites
*wR*(*F*^2^) = 0.121	H-atom parameters constrained
*S* = 1.06	*w* = 1/[σ^2^(*F*_o_^2^) + (0.0714*P*)^2^ + 1.0614*P*] where *P* = (*F*_o_^2^ + 2*F*_c_^2^)/3
2044 reflections	(Δ/σ)_max_ < 0.001
110 parameters	Δρ_max_ = 0.33 e Å^−^^3^
0 restraints	Δρ_min_ = −0.23 e Å^−^^3^

### Special details

**Table:**

Geometry. All esds (except the esd in the dihedral angle between two l.s. planes) are estimated using the full covariance matrix. The cell esds are taken into account individually in the estimation of esds in distances, angles and torsion angles; correlations between esds in cell parameters are only used when they are defined by crystal symmetry. An approximate (isotropic) treatment of cell esds is used for estimating esds involving l.s. planes.
Refinement. Refinement of *F*^2^ against ALL reflections. The weighted *R*-factor wR and goodness of fit *S* are based on *F*^2^, conventional *R*-factors *R* are based on *F*, with *F* set to zero for negative *F*^2^. The threshold expression of *F*^2^ > σ(*F*^2^) is used only for calculating *R*-factors(gt) etc. and is not relevant to the choice of reflections for refinement. *R*-factors based on *F*^2^ are statistically about twice as large as those based on *F*, and *R*- factors based on ALL data will be even larger.

### Fractional atomic coordinates and isotropic or equivalent isotropic displacement parameters (Å^2^)

**Table:**

	*x*	*y*	*z*	*U*_iso_*/*U*_eq_	
N1	0.07952 (5)	0.04557 (10)	0.74621 (8)	0.0160 (2)	
N2	0.19413 (5)	0.35854 (10)	0.82811 (8)	0.0188 (2)	
C1	0.22757 (6)	0.47046 (13)	0.77554 (10)	0.0214 (3)	
H1	0.2635	0.5294	0.8252	0.026*	
C2	0.21265 (6)	0.50475 (13)	0.65292 (10)	0.0212 (3)	
H2	0.2368	0.5865	0.6199	0.025*	
C3	0.16173 (7)	0.41697 (13)	0.57979 (10)	0.0233 (3)	
H3	0.1504	0.4372	0.4953	0.028*	
C4	0.12752 (6)	0.29891 (12)	0.63160 (10)	0.0208 (3)	
H4	0.0929	0.2362	0.5831	0.025*	
C5	0.14487 (6)	0.27409 (11)	0.75622 (9)	0.0154 (2)	
C6	0.10752 (6)	0.14970 (11)	0.81635 (9)	0.0160 (2)	
C7	0.10754 (7)	0.16392 (14)	0.95159 (10)	0.0269 (3)	
H7A	0.0734	0.2443	0.9679	0.040*	
H7B	0.1582	0.1873	0.9909	0.040*	
H7C	0.0910	0.0694	0.9838	0.040*	
C8	0.03861 (5)	−0.08031 (11)	0.78929 (9)	0.0151 (2)	
H8	0.0327	−0.0650	0.8766	0.018*	
C9	0.08258 (6)	−0.22419 (11)	0.77538 (9)	0.0169 (2)	
H9A	0.0978	−0.2272	0.6931	0.020*	
H9B	0.1285	−0.2242	0.8354	0.020*	
C10	0.03633 (6)	−0.36338 (12)	0.79419 (10)	0.0188 (3)	
H10A	0.0656	−0.4542	0.7812	0.023*	
H10B	0.0248	−0.3653	0.8787	0.023*	

### Atomic displacement parameters (Å^2^)

**Table:**

	*U*^11^	*U*^22^	*U*^33^	*U*^12^	*U*^13^	*U*^23^
N1	0.0176 (4)	0.0139 (4)	0.0170 (4)	−0.0002 (3)	0.0038 (3)	0.0006 (3)
N2	0.0187 (5)	0.0183 (5)	0.0191 (4)	−0.0021 (3)	0.0020 (3)	−0.0008 (3)
C1	0.0197 (5)	0.0199 (6)	0.0239 (5)	−0.0045 (4)	0.0007 (4)	−0.0011 (4)
C2	0.0208 (5)	0.0178 (5)	0.0253 (6)	−0.0030 (4)	0.0040 (4)	0.0038 (4)
C3	0.0283 (6)	0.0224 (6)	0.0186 (5)	−0.0045 (4)	0.0011 (4)	0.0038 (4)
C4	0.0244 (6)	0.0186 (5)	0.0184 (5)	−0.0052 (4)	0.0002 (4)	0.0000 (4)
C5	0.0154 (5)	0.0127 (5)	0.0184 (5)	0.0017 (4)	0.0039 (4)	−0.0007 (4)
C6	0.0155 (5)	0.0157 (5)	0.0173 (5)	0.0013 (4)	0.0039 (4)	0.0001 (4)
C7	0.0362 (7)	0.0279 (6)	0.0176 (5)	−0.0114 (5)	0.0075 (5)	−0.0030 (4)
C8	0.0179 (5)	0.0134 (5)	0.0140 (4)	−0.0012 (4)	0.0027 (4)	0.0004 (4)
C9	0.0176 (5)	0.0152 (5)	0.0179 (5)	0.0008 (4)	0.0023 (4)	0.0010 (4)
C10	0.0215 (6)	0.0136 (5)	0.0211 (5)	0.0015 (4)	0.0020 (4)	0.0019 (4)

### Geometric parameters (Å, °)

**Table:**

N1—C6	1.2726 (14)	C6—C7	1.5075 (15)
N1—C8	1.4623 (12)	C7—H7A	0.9800
N2—C5	1.3422 (14)	C7—H7B	0.9800
N2—C1	1.3432 (14)	C7—H7C	0.9800
C1—C2	1.3856 (16)	C8—C9	1.5304 (14)
C1—H1	0.9500	C8—C8^i^	1.5392 (19)
C2—C3	1.3833 (16)	C8—H8	1.0000
C2—H2	0.9500	C9—C10	1.5288 (14)
C3—C4	1.3874 (15)	C9—H9A	0.9900
C3—H3	0.9500	C9—H9B	0.9900
C4—C5	1.3940 (15)	C10—C10^i^	1.526 (2)
C4—H4	0.9500	C10—H10A	0.9900
C5—C6	1.5039 (14)	C10—H10B	0.9900
			
C6—N1—C8	122.56 (9)	H7A—C7—H7B	109.5
C5—N2—C1	117.45 (9)	C6—C7—H7C	109.5
N2—C1—C2	123.65 (10)	H7A—C7—H7C	109.5
N2—C1—H1	118.2	H7B—C7—H7C	109.5
C2—C1—H1	118.2	N1—C8—C9	108.70 (8)
C3—C2—C1	118.33 (10)	N1—C8—C8^i^	105.88 (7)
C3—C2—H2	120.8	C9—C8—C8^i^	112.63 (6)
C1—C2—H2	120.8	N1—C8—H8	109.8
C2—C3—C4	119.06 (10)	C9—C8—H8	109.8
C2—C3—H3	120.5	C8^i^—C8—H8	109.8
C4—C3—H3	120.5	C10—C9—C8	111.64 (8)
C3—C4—C5	118.76 (10)	C10—C9—H9A	109.3
C3—C4—H4	120.6	C8—C9—H9A	109.3
C5—C4—H4	120.6	C10—C9—H9B	109.3
N2—C5—C4	122.72 (10)	C8—C9—H9B	109.3
N2—C5—C6	116.91 (9)	H9A—C9—H9B	108.0
C4—C5—C6	120.37 (9)	C10^i^—C10—C9	110.46 (7)
N1—C6—C5	115.68 (9)	C10^i^—C10—H10A	109.6
N1—C6—C7	128.08 (10)	C9—C10—H10A	109.6
C5—C6—C7	116.23 (9)	C10^i^—C10—H10B	109.6
C6—C7—H7A	109.5	C9—C10—H10B	109.6
C6—C7—H7B	109.5	H10A—C10—H10B	108.1

Symmetry codes: (i) −*x*, *y*, −*z*+3/2.
